# A national evaluation of Project Cautioning And Relationship Abuse (‘CARA’) awareness raising workshops for first time offenders of domestic violence and abuse: protocol for a concurrent mixed-methods evaluation design

**DOI:** 10.3310/nihropenres.13609.1

**Published:** 2024-08-01

**Authors:** Sara A Morgan, Steph Scott, Joht Chandan, Rachel Armitage, David Culliford, Kate Jolly, Ruth McGovern, William McGovern, Jessica Roy, Rasiah Thayakaran, Tracey A Young, Julie Parkes

**Affiliations:** 1 Centre for Population Health Sciences, University of Southampton, South Academic Block, Southampton General Hospital, England, SO16 6YD, UK; 2 Population Health Sciences Institute, Newcastle University, Newcastle upon Tyne, England, NE1 4LP, UK; 3 Institute of Applied Health Research, University of Birmingham, University of Birmingham, England, B15 2TT, UK; 4 Birmingham Health Partners, Birmingham, B15 2TT, UK; 5 University of Huddersfield, Huddersfield, England, HD1 3DH, UK; 6 NIHR Applied Research Collaboration Wessex, School of Health Sciences, University of Southampton, Southampton, England, SO17 1BJ, UK; 7 Department of Social Work, Education and Community Wellbeing, Northumbria University, Newcastle upon Tyne, England, NE7 7QA, UK; 8 School for Policy Studies, University of Bristol, Bristol, England, BS8 1TZ, UK; 9 Sheffield Centre for Health and Related Research, School of Medicine and Population Health, The University of Sheffield, Sheffield, England, S1 4DA, UK

**Keywords:** Domestic violence and abuse, diversionary cautions, prevention, perpetrator workshops

## Abstract

**Introduction:**

Interventions related to the perpetration of Domestic Violence and Abuse (DVA) have gained traction over the past several years, in response to dissatisfaction by victims, an inadequate response from the criminal justice system, increased demand on police time and a lack of rehabilitative responses to the perpetration of domestic abuse. The CARA model is a conditional diversionary caution, offered by police for first time offenders of ‘standard’ or ‘medium risk’ domestic abuse, that engages perpetrators in awareness raising workshops and signposts them onto further services. Although quasi-experimental studies have indicated that CARA showed promise at reducing reoffending, the CARA model has yet to be evaluated nationally and there is no qualitative evidence related to understanding or learning about the lived experience of perpetrators and victims as they engage with the intervention.

**Methods:**

Using a concurrent pragmatic mixed methods design model we will undertake a national evaluation of CARA by triangulating quantitative data from up to nine police forces, and routine data from service providers, with qualitative data from workshop participants, victims and professional stakeholders to: (1) understand the long-term impact of CARA implementation on DVA reoffending and engagement with services and (2) explore perceptions and experiences of both delivery and receipt of CARA. We will use qualitative methodologies that draw on interpretivist and phenomenological perspectives, as well as quantitative methodologies using interrupted time series models, Poisson regression models, Geo mapping and a cost benefits analysis.

**Ethics and dissemination:**

Where currently the CARA model is being introduced as a national option for standard risk first-time offending, we will engage with policymakers and academics nationally in the live debate on its effectiveness and suitability during its roll-out. Ethical approval was approved by the University of Southampton on the 1 ^st^ June 2022 (Ref: ERGO ID: 71818.A1).

## Plain Language Summary

Over 2 million incidents relating to domestic violence and abuse (DVA) were reported to the police in England and Wales in the year leading up to March 2023. DVA leads to poor health and social outcomes for both victims and perpetrators. Consultation suggests that the system is struggling to support victims of domestic abuse and prevent repeat offending. Hampshire were keen to test an intervention that would improve outcomes for victims and their families. They developed a conditional diversionary caution called CARA offered by the police to those who have committed a crime related to domestic violence and abuse for the first time, where the incident was considered as ‘standard’ according to a domestic abuse risk checklist and professional judgement. Offenders are required to undertake two mandatory workshops that increase awareness of their behaviour and the safety of partners and children. They are further signposted onto services that support improvements in their health and social care needs that may contribute towards their offending behaviour. CARA cautions are now being offered across several regions. However, we don’t understand how offenders and victims feel about this intervention and we don’t know whether engagement in CARA leads to change in abusive behaviours over a longer period, such as 12 months after the intervention. We will interview offenders, victims and those involved in delivering CARA. We will aim to understand what worked and what didn’t work. We will look at what happens to offenders and the costs associated with this and examine whether there may be any differences in outcomes for those of different ethnic backgrounds or from different areas. To develop this plan, we consulted with both victim and offender groups. We will consult with both groups to improve our methodology, data collection and how we share our results with the public.

## Introduction

Domestic violence and abuse (DVA) is a grave human rights violation and represents a global public health epidemic ( Alhabib *et al.*, 2010; Ellesberg & Heise, 2005; Joachim, 2000), resulting in increased use of health services and vast social and economic costs ( Oliver *et al*., 2019). DVA is associated with a range of negative health and social outcomes including chronic pain, physical disability, poorer reproductive health, acquisition of HIV or other STIs, substance misuse and can also lead to death ( WHO, 2013). DVA is also linked to the exacerbation of mental health issues including PTSD symptoms, anxiety, and depression ( Ferrari *et al.*, 2014). There is now a strong body of empirical evidence demonstrating that, across a wide range of settings, women and girls are at more risk of violence perpetrated by an intimate partner than anyone else ( Oram *et al.*, 2022). Thus, whilst women are not the sole victim-survivors of DVA nor is it exclusive to heterosexual intimate relationships, they do represent the majority of victim-survivors worldwide. Current prevalence data suggests that over 2 million incidents relating to DVA in England and Wales occurred over a one-year period leading up to March 2023, with under half recorded as criminal offences ( ONS, 2023). These figures are likely to be a significant underestimate due to limitations of data capture methods. It is challenging to accurately reflect and measure the true prevalence of domestic violence given the secrecy and stigma which continues to surround it.

Measures and interventions that aim to reduce prevalence and/or the harms associated with DVA have gained traction over the last few decades, including the adoption of a public health approach similar to the approach taken to the management of physical violence within cities ( Chandan *et al*., 2020). However, there has also been a sustained reduction in capacity across the criminal justice system (CJS) at a time of increasing demand ( McConville & Marsh, 2020). Stakeholder consultation suggests that the CJS is struggling to constructively support victim-survivors, deter offenders or reduce reoffending, by means of an early intervention. Victim-survivors report feeling a lack of justice in formal systems dealing with DVA ( Hester *et al.*, 2023) and, collectively, professionals feel that they could be more effective. Furthermore, recent evidence suggests that COVID-19 has had a huge impact on the CJS, creating a backlog within an already pressurised process. Extreme delays within the CJS can only exacerbate the issues already present prior to the pandemic.

One innovative approach to diversion from the CJS pathway, “Project CARA”, was developed in 2011, as a conditional caution offered by the police to first time adult offenders of DVA of standard risk according to the DASH domestic abuse checklist and professional judgement. This initiative provided an alternative beyond a ‘simple caution’ or ‘no further action.’ A caution requires the offender to admit culpability in order to be an available disposal option. Hampshire Constabulary in England were ambitious to test an alternative response via an early intervention that would improve outcomes for victim-survivors and their families. Under this model, offenders are required to undertake two mandatory workshops that increase awareness of their abusive behaviour and the safety of partners and children. If, however, they fail to comply with the conditions of this caution, they may be prosecuted for the original offence. In contrast to restorative justice, CARA is an awareness raising intervention for offenders, that utilises a trauma-informed approach and motivational interviewing techniques. In these workshops, offenders are further signposted onto services that support improvements in the wider determinants of their offending behaviour, such as to primary care, drug and alcohol services or onto a community perpetrator programme. Project CARA itself rests on several assumptions about DVA and health inequalities. First, drawing on Marmot *et al.*, Project CARA recognises the potential lifelong impacts of DVA on health, social and economic outcomes throughout the life-course and aims to tackle the multiplicity of disadvantage and the impact that this can have upon health. Project CARA stresses the importance of early intervention and an understanding that health must not be viewed in isolation, both of which are at the heart of local Health and Wellbeing Board strategies ( Myhill & Bartlett, 2024). An initial experimental trial of CARA was undertaken in Southampton custody suite, showing a reduction in the frequency of re-arrest and prevalence of domestic abuse in the intervention arm, one year following allocation ( Strang *et al.*, 2017). No further evaluation has been conducted, and both national and qualitative evidence is lacking. Overall the literature is lacking in qualitative evidence around what works for perpetrators, including how to engage them in such interventions, and whether engagement leads to self-awareness and behavioural change. Despite this, CARA has been rolled out nationally and is likely to remain the main-stay intervention for eligible individuals.

## Aims and objectives

Using a pragmatic concurrent mixed methods design the aim of our study is to evaluate Project CARA following its wider rollout nationally. By using this approach to mixed method study design, both methods will be conducted and analysed independently before findings are later integrated together ( Creswell & Plano Clark, 2018).

Our research objectives are to:

- Understand the perceptions and experiences of clients, partners/former partners and professionals involved in the delivery of Project CARA.
- Examine whether the completion of the CARA conditional caution leads to a reduction in the incidence and/or severity of DVA at the population level.
- Develop an overarching logic model for Project CARA in order to understand the mechanisms of action of intervention components and anticipated outcomes (all phases).
- Explore the wider contextual factors around offending behaviour and answer research questions around the implementation of CARA including, for example, the types of violence that result in a CARA caution.
- Analyse the cost-benefits of Project CARA in comparison with a matched cohort who do not receive Project CARA.

## Patient and Public Involvement

Patient and Public involvement has been undertaken since the conception of this research project. We initially consulted with public representative groups, including both victim-survivor and offender groups, to understand the initial research scope. At the funding stage, the research project proposal was first screened for potential funding eligibility by a PPI funding panel meeting. At this meeting the proposal was scored according to possible impact to the public. Throughout the research project, we will involve both victim-survivor and offender groups in our decision making from, for example, understanding how to ensure recruitment is feasible and acceptable to ensuring that the interview covers appropriate and relevant topics for the participants. We will also invite individuals from both groups to feedback on the plain English summary report, and to be involved in the dissemination strategy. At least one representative will be invited to co-present at a national or international conference. All members of the research team work across sectors involving underrepresented and marginalised communities including, victim-survivors and the justice-involved.

## Qualitative study

### Sampling and recruitment strategy

The qualitative study will involve the recruitment of three different population groups:

- Phase One: CARA workshop attendees or others, in matters when a simple caution/no further action was the disposal option (referred to collectively as ‘clients’). Where possible, the inclusion criteria for CARA will be used to ensure that participants are broadly comparable.
- Phase Two: The partner/ex-partner of clients (referred to as ‘victim-survivors’)
- Phase Three: Professionals and stakeholders comprising CARA facilitators, police staff, and third sector organisations (referred to as 'stakeholders'). It is anticipated that participants will span organisational structures, operational and strategic level roles

In phase 1 and 2, participants will be purposively sampled according to geographical location, socio-economic status, ethnicity, and CARA participation status. Whilst we will aim for gender diversity within our sample, we have not defined this as a primary sampling variable in recognition of the gendered nature of DVA.

Sample size will be guided by the breadth and focus of the research question(s), the demands placed on participants, the depth of data likely to be generated, and the analytic goals and purpose of the overall project. Following recommendations for pragmatic assumptions around sample size ( Braun & Clarke, 2019), it is anticipated that up to 15 clients, 15 victim-survivors and 15 stakeholders will be interviewed.

***Phase one***. Participants will be recruited from two separate cohorts: (1) a cohort recruited from those involved with or accessing Project CARA workshops, “CARA workshop attendees *”* within a participating police force area and (2), a cohort of broadly comparable participants without a Project CARA disposal option in a non-CARA participating area. It is anticipated that CARA workshop attendees will be recruited primarily through the [agency] who have responsibility for the design and delivery of Project CARA workshops within the following participating police force areas: Avon & Somerset, Cambridgeshire, Dorset, Hampshire, Leicestershire, Norfolk, Thames Valley, West Midlands, and West Yorkshire. All participants from non-CARA participating areas will be recruited with the support of police staff and third sector organisations who will act as gatekeepers for the study.

***Phase two***. In-depth qualitative interviews will be undertaken with victim-survivors at two time-points. Interview one will take place within three months of their partner’s/former partner’s disposal (e.g. CARA attendance or simple caution/no further action) and interview two will take place six months later.

***Phase three***. Stakeholders will be contacted directly by the research team to take part in an interview or focus group session. We will use purposive sampling to ensure a range of individuals are recruited across different geographical location, job roles (including police, commissioners, facilitators and managers and voluntary sector organisations who provide support in the DVA sector) and gender.

### Approach to data collection

It is anticipated that, where possible, interviews will take place in person due to safeguarding and privacy concerns. Nevertheless, each interview will be considered and discussed by the research team, with remote interviews considered on a case-by-case basis where necessary. Should a victim-survivor and client with an established relationship both agree to be interviewed, different researchers will interview each party. Interviews will be steered by a topic guide but remain flexible to reflect the experiences, perspectives, and authentic voice of each participant. For clients and victim-survivor interviews, the interview is anticipated to cover topics including biographic detail, history of primary relationship, circumstances of offence, experiences of trauma and stigma, contact with police, CARA experience, outcomes, support, and changes over time points in mental and physical health and wellbeing. For phase 3, interviews and focus groups will be conducted remotely, using an appropriate video-conferencing platform, with professionals and stakeholders comprising CARA facilitators and managers, police staff and third sector organisations, to explore and evaluate facilitators and barriers to the implementation of CARA. This phase comprises both one-to-one interviews and focus groups as it is anticipated that professionals may prefer to take part on an individual basis. Interviews and focus groups will be semi-structured and guided by a topic guide. This will cover organisational approaches to DVA, CARA and non-CARA disposal outcomes, wider contextual factors around offending behaviour and barriers and facilitators to reducing DVA and associated physical and mental health impacts.

All participants will be provided with a participant information sheet. Time will be allocated at the beginning of the interview to reiterate the points outlined in the information sheet, and make sure that participants do not have unanswered questions. Participants will then be asked to complete and sign a consent form. A period of debriefing will be built into the end of all interviews and focus group sessions where required and researchers will carry information with them to signpost interviewees to further support and information.

***Staged consent***. It is anticipated that consent for Phase 1 and Phase 2 participants will be obtained using a two-stage process. First, facilitators from the [agency] will initiate contact between the research team and prospective participants. Stage 1 consent will therefore comprise permission for the [agency] to pass individuals’ contact details to the researchers and with an understanding that they will be contacted about the study. Details about the study will be summarised in a short leaflet or flyer. Permission for the researchers to telephone and text participants will be explicitly obtained. Contact details will be shared between the [agency] and the researcher via a spreadsheet shared through the password protected University drop-off system. The researchers will subsequently attempt to contact participants within two weeks of Stage 1 and provide them with further information about the study (including a full participant information sheet). If participants agree to take part, a date/ time for interview based on their convenience and wishes will be agreed.

Stage 2 consent will involve the allocation of time at the beginning of the interview to check that participants have read the participant information sheet and make sure all queries have been answered. If participants decide to proceed, full consent for participation in the study will be obtained. For participants not involved with or accessing Project CARA, this two-phase approach to obtaining consent will be replicated using police leads or third sector organisations as gatekeepers.

## Ethics and governance

Research ethical approval has been sought from the University of Southampton (Higher Education Institute) Ethical Committee and approved on the on the 1 ^st^ June 2022 (Ref: ERGO ID: 71818.A1) As this research study is not being conducted within prisons nor within the national health service, neither HM Prison and Probation Service or Health Research Authority approval will be needed, respectively. Upholding ethics, good governance and quality in practice is central to all processes. The project will also comply fully with the ESRC's Research Ethics Framework, The Offender Health Research Network and GDPR. This includes upholding the ethical research governance in line with the Ethics Policy Statement at the University of Southampton https://www.southampton.ac.uk/about/governance/policies/ethics.page.

There are a number of central tenets that should be maintained throughout the research. These include the moral principles guided by four main principles of bioethics ( Beauchamp & Childress, 2013). The principles are autonomy (informed consent), non-maleficence (do no harm), positive beneficence (benefits of research outweigh the risks) and justice (research strategies and procedures are just and fair).

Police data will be shared as anonymised, aggregated form. Information sharing agreements have been developed and have been signed by the majority of force areas. No additional permissions are required to access this routine data. The lead researcher handling police data will undertake national police vetting with Warwickshire police in order to handle police data. Each police force will be required to develop, regularly review and sign a Data Protection Impact Assessment (DPIA) for the project.

### Consent and confidentiality for qualitative study

At the beginning of all interviews and focus groups, participants will be asked to provide informed, written and verbal consent. Study documentation will make the right to withdraw and voluntary nature of participation clear, describe limits to confidentiality and anonymity and highlight how data will be used. All participant focused information will be written in plain English. Interviews will be recorded using a digital audio recorder when conducted in person. When interviews are conducted via TEAMS video conferencing, the recording function will be used. Interview audio files will be transcribed verbatim into a Microsoft Word .docx file (Microsoft ^®^ Word for Microsoft 365 MSO (Version 2308) Free Microsoft 365 Online | Word, Excel, PowerPoint Interviews and focus groups will be transcribed externally using an approved transcription service. Audio recordings will be deleted once an accurate transcript has been produced. All anonymised interview data will be labelled with the unique participant identifier assigned during consent processes and will be stored in password-protected computers by the research team involved in data analysis. All personal data (e.g. name, contact details) will be confidential and will be kept securely and separately to anonymised research data. All personal data for the purposes of data collection, such as mobile numbers, will be kept until the follow-up interview. Three attempts to contact the participant (including text and phone calls) will be made, after which time all personal data will be deleted. Paper-based files will be stored in a locked filing cabinet. At the end of the study all anonymised files will be kept securely in the University’s repository for ten years.

Wherever possible, confidentiality will be assured and the study will have a comprehensive confidentiality and safeguarding protocol in place. However, if a participant discloses information that may mean the future harm of another individual, then the research team may need to share that information with relevant authorities. If such a scenario arose, the researcher would discuss with their supervisor to decide next steps. If there is an immediate risk of harm, then emergency services would be called. Information will be disclosed and confidentiality breached in the following circumstances:

1. When information given by the client concerns the abuse, harm or neglect of a child or when we have reason to believe that a child is being abuse, harmed or neglected. (This refers to any new cases and/or events).
2. If by keeping confidential the client or another person is likely to suffer serious injury.
3. If the Police have a court order for specific information relating to the client.
4. We are obliged to pass information to the relevant authorities if the information relates to the Prevention of Terrorism Act (1990) or Female Genital Mutilation (FGM Act 2015)
5. If the client discloses information relating to an offence either committed or planned.
6. If the client gives us any information that relates to unprofessional activity (for example illegal sale of drugs to other participants)

### Researcher safety

It is important to put appropriate safeguards in place for researchers. The qualitative component of this research study will be undertaken in accordance with Newcastle University’s lone working policy. It is anticipated that most interviews will be undertaken alone, and wherever possible will take place in-person. Researchers will be able to contact line managers out of hours as well as the university's security team. Researchers will have line manager support to debrief following interviews and focus groups. If undertaking interviews alone, before commencing interviews the interviewer will text message the appointed contact within the department prior to beginning the visit and immediately following the meeting. If the researcher does not make contact at the time indicated then the steps outlined in the lone working protocol will be carried out.

### Data analysis

This qualitative study will take a flexible interpretivist position, drawing on several methodological frameworks and approaches. Phases one and two are underpinned by Interpretative Phenomenological Analysis (IPA) ( Smith & Osborn, 2003). IPA seeks to appreciate and understand the participants perspectives through their self-interpretation and description of the lived experience of the phenomena studied ( Pietkiewicz & Smith, 2014) in this case DVA. Further, use of IPA aligns with a qualitative longitudinal approach in aiding exploration of how and why experiences and perceptions change over time, and by building in time for reflection on the part of both participant and researcher ( Graham-Wisener *et al*., 2019). Meanwhile, Phase three will draw on the constant comparative method through use of reflexive thematic analysis ( Campbell *et al*., 2021) which emphasizes reflexive engagement with theory, data and interpretation (Braun & Clarke, 2020).

All data will be imported using a qualitative research tool, such as open source Taguette. Transcripts will be coded line-by-line and then systematically indexed into data tables to generate detailed descriptive themes. These descriptive themes will be compared to identify patterns, similarities and differences in the data, and relationships between them elaborated, to generate analytical themes, and a consistent interpretation of the whole dataset. Themes will be discussed, considered and refined at regular project meetings with the wider project team, using a process defined as pragmatic double coding, whereby transcripts will be independently coded by at least two members of the research team, and discussed.

## Quantitative study

Within the quantitative study we will aim to:

1. Undertake a propensity score matched (PSM) cohort study to examine the individual level changes in recidivism and re-offending of DVA perpetrators.
2. Examine the population level effects in the incidence of DVA following the introduction of CARA.

### Population, intervention, comparison and outcome

**Figure 1.**
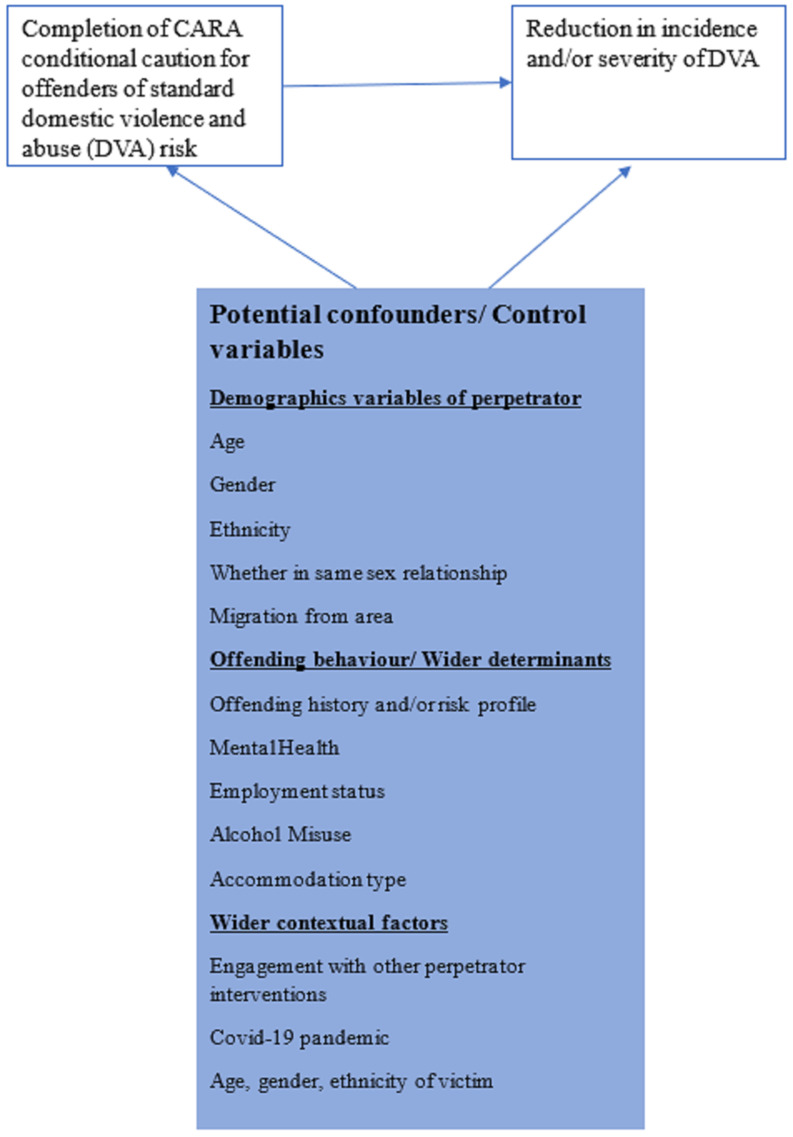
Hypothesis 1: Completion of the CARA conditional caution leads to a reduction in incidence and/or severity of DVA at the population level.

A previously published study examining the impact of the CARA was undertaken across the West Midlands ( University of Birmingham, 2021). As part of that project a PSM cohort approach was chosen to disentangle the treatment effects from confounding variables which may exist in this study ( Figure 1). However, as this study was only undertaken in one locality it was not sufficiently powered nor contained a diverse enough population to examine whether changes in recidivism or reoffending differed per offender sub-groups in the population such as by age, sex, ethnicity or socio-economic group. Undertaking such an evaluation of these sub-groups would be invaluable in providing further insight into specific groups of offenders who may respond better or worse to the CARA intervention. In this study, we will be able to do this as we are undertaking a nation-wide study to examine the effects of the interventions. Further details on the PSM cohort approach are below:

***Data source***: Data held on the police Record Management System (RMS) database, or equivalent, will be extracted, pseudonymised and shared in aggregated form.

***Population***: The population sample to be included in the analysis are those that are eligible for the CARA intervention.

***Intervention***: Those who have undertaken the CARA intervention will form the intervention group. CARA is a conditional diversionary caution offered by the police to first time adult offenders of DVA of standard risk or medium risk (in some areas). Offenders are required to undertake two mandatory workshops that increase awareness of their abusive behaviour and the safety of partners and children. In contrast to restorative justice, CARA is an awareness raising intervention for offenders, that utilises a trauma informed approach and motivational interviewing techniques across the pair of workshops. In these workshops offenders are further signposted onto services that support improvements in the wider determinants of their offending behaviour, such as to their GP, drug and alcohol services or onto a community perpetrator programme.

***Comparison***: Using PSM approaches a comparator group will be formed from those who meet the eligibility criteria for CARA but are based in an area that is currently not delivering the CARA intervention. Covariates for the matching will include demographics, such as age, gender, ethnicity, whether in same sex relationship; offending behaviour/wider determinants, such as offending history and/or risk profile, mental health, employment status, alcohol misuse, accommodation type and; wider contextual factors, such engagement with other perpetrator interventions.

***Outcome measures***: The primary outcome will be DVA reoffending within 12 months. The primary outcome will be described as either i) number of DVA arrests ii) number of DVA crimes. The secondary outcome(s) will include for example total number of arrests, crime severity score, total number of offences.

### Analysis of quantitative data

***Interrupted time series***. Using interrupted time series (ITS), we will also examine whether the introduction of the CARA conditional caution leads to a hypothesised population-based reduction in the incidence of DVA, as hypothesised in Figure 2. A secondary outcome will be to assess if there was a change in the severity of recorded DVA before and after the introduction of CARA.

 As an intervention study design, ITS is a valuable study design compared to traditional observational study designs (such as cohort or case control studies) particularly when there is a need for retrospective evaluations which have already been implemented, either without randomization or to a whole population, both of which are applicable in the CARA intervention ( Bernal *et al.*, 2017). By nature, ITS can be applied to natural experiments when an intervention is introduced at the population level at a clearly defined period of time and involves observing a single population over time.

In an ITS study, a time series of the outcome of interest (incidence of domestic violence and abuse) is used to establish an underlying trend, which is ‘interrupted’ by an intervention (e.g. CARA being offered in a force area) at a known point in time. The pre-intervention underlying trend is compared with the post-intervention period to identify whether the intervention is associated with any changes in outcome.

Initially summary statistics and plots will be undertaken, including a scatter plot of the time series demonstrating before and after comparisons of the incidence of DVA across the force areas. These will include sub-analyses, by age, gender, ethnicity, force area and whether in same sex relationship.

Following this a segmented regression model such as the one below will be constructed to undertake the ITS model:


Yt-=α+β1Tt+β2Xt+β3XtTt

Yt is the aggregated outcome variable measured at each equally-spaced time point (month)

Tt is time

Xt is study phase

XtTt is time after interruption

α, β1, β2 and β3 are model parameters

As our outcome measure (DVA occurrences) are count data, we will also undertake a Poisson model. Calendar quarters will be included as a categorical variable to account for seasonality. The final model will provide a measure of relative risk with 95% confidence intervals to describe the change in incidence in DVA across the CARA sites before and after the introduction of the intervention. We will account for covariates of interest in the final model to examine any changes specific to offender subgroups of interest, including age, sex, ethnicity and socioeconomic status. Confounding events or co-interventions that will affect study outcome will be explored through consultation with the research team, including all stakeholders, and through the qualitative research. Time varying confounders might include, for example, lockdowns from the COVID-19 pandemic.

Where possible we will also aim to undertake a controlled interrupted time series (CITS) ( Bernal *et al.*, 2018). In this case the control data will be derived from sites where CARA was not delivered during the study timeframe. The CITS model can be described as:


Yt-=α+β1Tt+β2Xt+β3XtTt+β4Zt+β5ZtTt+β6ZtXt+β7ZtXtTt

Yt is the aggregated outcome variable measured at each equally-spaced time point (month)

Tt is time

Xt is study phase

XtTt is time after interruption

Zt is treatment or control group

ZtTt is time for treatment and 0 for control,

ZtXt is study phase for treatment and 0 for control,

ZtXtTt is time after interruption for treatment and 0 for control

α, β1, β2 , β3 β4, β5, β6 and β7 are model parameters

***Geomapping***. We will also undertake geomapping using postcode data from regional and national data (either from the address where the incident took place or the perpetrator’s address). The postcode data will be input using googlemaps to generate a visual-coloured map. The visuals produced through this method are not accurate enough to render an individual’s home address to become identifiable; postcodes are replaced with colour patches, similar to heat maps, covering a large area. The postcodes entered into google through this process are not stored by google and therefore no data will be stored and shared through this method.

***Health economics analysis***. The aim of the health economic analysis will be to examine the cost-benefits of Project CARA with matched controls. Relevant benefits will include incidents of reoffending and quality adjusted life years (QALYs) obtained from the published literature. Cost considerations will include the cost of delivering Project CARA, including mobilisation and ongoing delivery costs and for both groups we will examine lost economic output, health, legal, social and specialist resources and any personal resource consequences (for example damages, divorce). The perspective of the analysis will include health and social care, criminal and legal and specialist services in the first instance and will explore the inclusion of costs to the victim in a wider cost perspective.

Data sources will include the CARA victim contact form, Crime Survey for England and Wales, and information collected from police taking part in the project. Unit costs will be taken from relevant sources such as the Home Office or Department of Health and Social Care. Results will be presented in a cost-benefit analysis as incremental costs per reoffence avoided and incremental costs per QALY. Probabilistic sensitivity analysis (PSA) will be used to allow for uncertainty around cost and effectiveness estimates.******

***Routine service data***. Routine data from the main third sector implementing agencies will be used to examine the fidelity, dose and reach of the CARA intervention. Routine data sources may include, for example, video observations of group work, referral forms and outcome questionnaires. These will be summarised descriptively. All quantitative data sources will be aggregated.

### Integration of findings

Dedicated time is built in at the end of the project for quantitative and qualitative data integration. It is anticipated that our approach to data integration will be twofold, first by ‘following a thread’ to allow each phase of work to inform the next, followed by formulation of a data matrix to illustrate patterns, surprises and paradoxes. ( Cathain *et al.*, 2010; Creswell & Clark, 2017) Both sets of data will be used to further enhance an overarching logic model for Project CARA in order to understand the mechanisms of action of intervention components and anticipated outcomes.

## Strengths and limitations of this study

- This is the first national study of Project CARA across several regional areas. We will evaluate the process, impact and cost benefit of Project CARA, as a novel and mainstay intervention for offending of domestic violence and abuse, across multiple sites.
- This will be the first-time qualitative methods will be used to examine the implementation and outcomes of CARA. This will lead to an in-depth and detailed understanding of the CARA model from the perspectives of victim-survivors, perpetrators and professionals. This will explore victim-survivors experiences of CARA as well as why perpetrators’ behaviour may change as a result of CARA involvement.
- In-depth qualitative data will be triangulated with quantitative data to provide a comprehensive and detailed analysis of the CARA model. Quantitative analysis will include an interrupted time series (ITS), to examine whether the introduction of the CARA diversionary caution leads to a hypothesised population-based reduction in the incidence of DVA nationally.
- The study is likely to be limited by the challenges associated with different aspects of data collection and engagement with the model. This may mean that not all police forces’ quantitative data is represented equally. We may be limited by the uptake of the CARA model, and the extent to which victim-survivors and perpetrators may be willing to engage with researchers.

### Dissemination plan

The main outputs from this research study will be a formal report and a short summary report in plain English (referencing the full report) outlining the key methods, results and implications for policy and practice. The target audience for this report will primarily be composed of individuals and organisations involved in health, social care and policing. This might include, for instance, volunteers, social care workers, investigators, researchers, and clinicians. As the audience may not be familiar with research, the language will be in plain English, with limited technical language relevant to the research. Further outputs include dissemination within academic and policy making circles. These have been described in the next section.

 The potential impacts of the research study are as follows:

- The research primarily will have an impact on vulnerable population groups (offenders and victim-survivors) who are not effectively engaged in research as they are harder to reach. The risk of not engaging this population group, however, will widen health inequalities and its impact on society.
- The research will have a long-term impact on the health and welfare of victim-survivors and children (in England and Wales) that are affected by DVA, a vulnerable population affected by multiple adversities.
- The research will provide new contextual evidence in the academic literature on the nature of offending behaviour for this population group.
- Through the development of the logic model, and the wider implementation plan, the research will inform national implementation of CARA through an understanding of the contextual factors arising from local environments.
- The health economic evaluation will provide information about the potential costs to the health and social care system in England and Wales, thereby informing decision making: on the cost-effectiveness of the intervention, the potential to make cost-savings and improve public finances.
- The results of the study will be shared with key parliamentary groups, including the Home Office, the Ministry of Justice, thereby influencing decision-making on the commissioning of services. The evidence may support policy decisions or changes to legislations, regulations and guidelines on the use of conditional cautions for domestic violence and abuse.
- It is expected that at least three publications will be published in academic journals including open access journals. This will be to maximise the evidence-base and knowledge uptake across academic disciplines; and will benefit the wider academic community in the future.
- Where the stakeholders of the research are also non-governmental organisations and public services, the research will also increase the effectiveness of public services and policy.

## Data Availability

No data are associated with this article.
