# Supplementary material for: A national evaluation of Project Cautioning And Relationship Abuse (‘CARA’) awareness raising workshops for first time offenders of domestic violence and abuse: protocol for a concurrent mixed-methods evaluation design
Source: NIHR Open Res. 2024 Aug 1;4:43. [Version 1] doi: 10.3310/nihropenres.13609.1 (PMC11474160; doi:10.3310/nihropenres.13609.1)
Supplement: Peer review history

# Reviewer report: Reviewer response for version 1

**DOI:** 10.3310/nihropenres.14774.r33056

**Published:** 2024-10-14

**Authors:** Chris Dyke

**Affiliations:** 1 Kingston University, London, England, UK

This is a comprehensive protocol setting out a valuable study to test the effectiveness of a DVA intervention, addressing what the authors correctly identify as a major gap in the knowledge base for DVA practitioners and researchers. The details of this evaluation design are clear and seem suited to the aims and objectives involved.

 I have made some relatively minor points below for consideration in terms of honing this particular document, but I hope that this important piece of research can start shortly - I wish the researchers the very best of luck.

 Introduction: the context is spelled-out clearly, with some recent background data from ONS. At the same time, the use of research around the health and socioeconomic costs of DVA, while relevant, could both be more specific and more up-to-date: it shouldn't be difficult to find more recent evidence with more quantified findings around the multiple impacts of DVA on an individual and societal level (I'm conscious that this project has been in the works for some time already, so what was originally an up-to-date background section may need updating). The 'recent evidence' about the impact of COVID on the CJS needs to be cited.

 DASH needs to be spelled-out and explained (and critiqued).

 The description of CARA is interesting but generally unreferenced - needs more sources. Date for Marmot et al?

 Data collection for Qualitative study: the approach seems thorough and suitable, though it would be interesting to see a bit of reflection/discussion of why this approach was selected, and drawing upon what kind of evidence or similar studies.

 Quantitative study: it may be a stylistic point, but while you've laid out full details of the study, I had to go back over it a couple of times - it might be worth spending a couple of sentences spelling out what the quantitative study will do (as well as its aims) right at the start of this section. This would help a topic specialist unfamiliar with the method used (as many readers, including this one, might be) to grasp a general understanding of what you're doing in the quantitative study before you go into the (comprehensive) details.

Is the study design appropriate for the research question?

Yes

Is the rationale for, and objectives of, the study clearly described?

Yes

Are sufficient details of the methods provided to allow replication by others?

Yes

Are the datasets clearly presented in a useable and accessible format?

Not applicable

Reviewer Expertise:

Domestic Violence in the context of social work, criminology and psychology; decision-making around domestic violence; mixed methods design (though I am not familiar with all of the techniques used in this protocol).

I confirm that I have read this submission and believe that I have an appropriate level of expertise to confirm that it is of an acceptable scientific standard.

# Reviewer report: Reviewer response for version 1

**DOI:** 10.3310/nihropenres.14774.r32639

**Published:** 2024-09-05

**Authors:** N Zoe Hilton

**Affiliations:** 1 Waypoint Centre for Mental Health Care, Waypoint Research Institute, Penetanguishene, Ontario, Canada

**Summary**

 This protocol lays out the rationale, objectives, methods, dissemination plans and implications of a multi-site evaluation of a domestic violence/abuse offender diversion program, helping to close a knowledge gap about the reception and effectiveness of this national program. This includes qualitative research with offenders, victims, and service providers, and quantitative research comparing individuals eligible for/completing CARA matched with individuals in regions where CARA is not available.

**Is the rationale for, and objectives of, the study clearly described?**

 Yes

**Is the study design appropriate for the research question?**

 Yes, mostly so.

 The design is appropriate to test the hypothesis of whether completion of CARA is associated with a reduction in incidence/severity of DVA, But perhaps not the stronger hypothesis that is stated as H1, that CARA "leads to" a reduction in DVA.

 In the quantitative study, the Population is described as "those that are eligible for the CARA intervention" but under Intervention it is stated that "Those who have undertaken" CARA will be the intervention group, suggesting that only data from CARA completers will be included in the treatment group. An intent-to-treat design would be more valuable because treatment completers are lower risk, as attrition from intervention tends to be related to risk of DVA.

 Consider including all individuals eligible for CARA and adding to the potential confounders/control variables the extent of engagement/completion of CARA.

 It is stated that matching includes offending history and/or risk profile - when would risk profile not be used? Is the DASH available and conducted in non-CARA regions? Depending on how well the DASH has been shown to identify risk of subsequent DVA, it could be used as a risk baseline to determine how well CARA reduces the expected level of DVA recidivism based on DASH risk levels. Or if the risk assessment has limited validity, this would have the potential to underestimate/overestimate the benefit of CARA if individuals identified as lower risk were actually at relatively high risk of subsequent DVA when referred to CARA, or those assessed at moderate risk were actually relatively low risk.

**Are sufficient details of the methods provided to allow replication by others?**

 Yes

**Are the datasets clearly presented in a useable and accessible format?**

 Not applicable

Is the study design appropriate for the research question?

Partly

Is the rationale for, and objectives of, the study clearly described?

Yes

Are sufficient details of the methods provided to allow replication by others?

Yes

Are the datasets clearly presented in a useable and accessible format?

Not applicable

Reviewer Expertise:

Domestic violence, risk assessment

I confirm that I have read this submission and believe that I have an appropriate level of expertise to confirm that it is of an acceptable scientific standard.
